# Low molecular weight heparin decreases mortality and major complication rates in moderately severe and severe acute pancreatitis–a systematic review and meta-analysis

**DOI:** 10.3389/fmed.2023.1241301

**Published:** 2023-10-25

**Authors:** Cristina Patoni, Stefania Bunduc, Levente Frim, Dániel Sándor Veres, Fanni Dembrovszky, Anna Júlia Éliás, Dániel Pálinkás, Péter Hegyi, Bálint Mihály Erőss, Péter Jenő Hegyi

**Affiliations:** ^1^Center for Translational Medicine, Semmelweis University, Budapest, Hungary; ^2^Carol Davila University of Medicine and Pharmacy, Bucharest, Romania; ^3^Central Military Emergency Hospital Dr. Carol Davila, Bucharest, Romania; ^4^Fundeni Clinical Institute, Bucharest, Romania; ^5^Institute for Translational Medicine, Medical School, University of Pécs, Pécs, Hungary; ^6^Department of Biophysics and Radiation Biology, Semmelweis University, Budapest, Hungary; ^7^Doctoral School of Health Sciences, Semmelweis University, Budapest, Hungary; ^8^Department of Gastroenterology, University of Military Hospital–State Health Centre, Budapest, Hungary; ^9^Division of Pancreatic Diseases, Heart and Vascular Center, Semmelweis University, Budapest, Hungary; ^10^János Szentágothai Research Center, University of Pécs, Pécs, Hungary

**Keywords:** acute pancreatitis, anticoagulants, low-molecular-weight-heparin, meta-analysis, guideline

## Abstract

**Background:**

Routine anticoagulation therapy in acute pancreatitis (AP) is not recommended by the guidelines in the field, although it is frequently used in clinical practice.

**Objectives:**

We aimed to analyze the efficacy and safety of adding anticoagulants therapy to AP management.

**Methods:**

The systematic search was performed in three databases on the 14th of October 2022 without restrictions. Randomized controlled trials (RCTs) and observational studies that reported the differences in the outcomes of AP for patients receiving anticoagulants (intervention group) in addition to the standard of care (SOC), compared to patients managed by SOC alone (control group), were eligible. A random-effects model was used to calculate the pooled odds ratios (OR) and mean differences (MD) with the corresponding 95%-confidence intervals (CI). We performed subgroup analysis for study design and disease severity, among other criteria.

**Results:**

Of the 8,223 screened records, we included eight in the meta-analysis. Except one, all studies reported on low-molecular-weight heparin (LMWH). Both RCTs and observational studies reported results in favor of the LMWH group. Subgroup RCTs’ analysis revealed significantly decreased odds of mortality [OR 0.24; 95%CI 0.17–0.34] and multiple organ failure [OR 0.32; 95%CI 0.17–0.62] in the intervention group. Moreover, the need for endoscopic or surgical interventions [OR 0.41; 95%CI 0.28–0.61] were significantly reduced by LMWH. The subgroup analyzes for moderate and severe cases, respectively, yielded similar results. Due to limited data, we could no perform subgroup analysis for mild cases.

**Conclusion:**

LMWH therapy reduces major complication rates in moderate and severe AP. Across all identified RCTs, LMWH were initiated early after AP diagnosis and improved its prognosis.

## Highlights

1. Summarize the established knowledge on this subject
  - There is a need in pancreatology for novel treatment approaches to decrease the severity and mortality of acute pancreatitis (AP).
  - The existing AP guidelines do not provide any guidance about Anticoagulant therapy in AP.
2. What are the significant and/or new findings of this study?
  - LMWH can significantly decrease mortality, multiple organ failure, and the need for endoscopic or surgical intervention rates in AP.
  - The prophylactic and therapeutic doses of low molecular weight heparin are both effective.
  - Data about mild AP are scarce.

## Introduction

Acute pancreatitis (AP) has an increasing incidence globally that has risen by 30% in recent decades (1, 2). Moreover, it is among the leading causes of emergency gastroenterology department admissions in Europe (3) and United States (4) and is associated with a substantial overall mortality rate of 5%, reaching up to 30% in severe cases (5). Therefore, finding new therapeutic methods for the reduction of severity and mortality of AP is an unmet need in pancreatology (6).

Anticoagulants therapy for the management of AP is based on the expertise of the attending physicians, and there are no recommendations despite it being frequently utilized in clinical practice (5). However, the decision to initiate systemic anticoagulation can be challenging due to the often unpredictable course of AP, which is associated with an increased risk of hemorrhage (7) or may require endoscopic or surgical treatment.

A few randomized controlled trials (RCTs) revealed decreased major complication rates after adding anticoagulants therapy to the standard of care (SOC) for the management of acute AP (8, 9). Moreover, a meta-analysis that evaluated the effectiveness of anticoagulants in severe AP cases (10) confirmed that it can significantly improve disease prognosis.

Our systematic review and meta-analysis aimed to investigate the efficacy and safety of adding anticoagulants to the SOC for managing AP across all disease severity stages. We analyzed moderately severe and severe cases, the anticoagulant dose, the duration of therapy, and pooled separately the results of the RCTs.

## Materials and methods

This systematic review and meta-analysis was performed according to Cochrane recommendations (11) and is in accordance with the Preferred Reporting Items for Systematic Reviews and Meta-Analyzes (PRISMA 2020) Statement (Supplementary Table S1) (12). Methods of the analysis and inclusion criteria were established in advance and documented on the International Prospective Register of Systematic Reviews (PROSPERO; CRD42021283239). Besides the reported protocol, we performed additional subgroup analyzes according to the study design, disease severity, treatment dose, and therapy duration.

### Systematic search

To formulate our clinical question and identify the eligible studies, we used the population-intervention-control-outcome (*PICO*) framework. Our investigated population (*P*) consisted of patients diagnosed with AP. Eligible studies compared the outcomes of AP in patients who received anticoagulant treatment in addition to the standard of care for disease management (*I*) to patients who did not receive anticoagulant treatment (*C*). Our primary outcome (*O*) was the in-hospital mortality rate. Our secondary outcomes were: length of hospital stay (LOH), local and systemic complications occurrence (multiple organ failure (MOF), acute kidney injury (AKI), acute respiratory distress syndrome (ARDS), pancreatic necrosis, pseudocysts), the need for endoscopic or surgical interventions, progression of AP severity, thrombotic or bleeding events.

The systematic search covered three databases: MEDLINE (via PubMed), EMBASE, and Cochrane Central Register of Controlled Trials (CENTRAL), from inception until the 14th of October 2022.

Our search strategy is detailed in the Supplementary material (see Appendix 1). The query included terms for all types of anticoagulants and acute pancreatitis. We did not use other restrictions or filters during the search.

### Selection and eligibility

Besides the PICO criteria, we considered eligible RCTs and observational studies. Other study designs were not eligible for our review. Also, animal studies were excluded. Both peer-reviewed reports available as *in extenso* published papers and conference abstracts were considered for analysis, without language restrictions. The pool of articles provided by the advanced search was downloaded to Endnote X (Clarivate Analytics, Philadelphia, PA, United States). Duplicates were removed first automatically and then manually. The selection was made by 2 independent review authors (CP and AÉ), based first on the title and abstract, and subsequently on full-text contents Cohen’s Kappa coefficient was calculated to evaluate inter-rater agreement during each selection step and (13) disagreements were resolved by a third independent investigator (SB) (Detailed in Appendix 2).

### Data collection

Data was extracted from each eligible article and collected into a standardized Excel (Microsoft Corporation, Redmond, Washington, United States) datasheet (Detailed in Appendix 3).

### Statistical analysis

Whenever one outcome was reported in a minimum of three studies, a meta-analysis was performed, and forest plots were used to visualize graphically the results. We anticipated considerable between-study heterogeneity, so a random-effects model was used to pool effect sizes. For continuous outcomes, we calculated mean differences (MD). For dichotomous outcomes, we calculated odds ratios (OR) with corresponding 95% confidence intervals (CI) to investigate the differences between the two groups (intervention vs. comparison; Detailed in Appendix 4). We assessed the between-study heterogeneity by I (2) statistics (14). We performed subgroup analyzes according to the study design, disease severity, treatment dose, and therapy duration. For the subgroup analysis, we used a fixed-effects “plural” model (Detailed in Appendix 5).

Outlier and influence analyzes were carried out using the leave-one-out method and influential plots to assess the robustness of the conclusions (15, 16). Publication bias was evaluated by Egger’s test and visual inspection of funnel plots (Detailed in Appendix 6).

For meta-analysis, we used the meta (Schwarzer 2022, v5.2.0) (17) and dmetar (Cuijpers, Furukawa, and Ebert 2020, v0.0.9000) (18) packages of R software (19).

### Risk of bias assessment

The quality assessment of the eligible reports was carried out by two independent reviewers (CP and LF), and disagreements were solved by third-party arbitration. We used the Revised Cochrane risk-of-bias tool for randomized trials (RoB 2) to assess RCTs (20). The Risk Of Bias In Non-randomized Studies-of Interventions (ROBINS-I) tool was used for the observational studies (21) (Detailed in Appendix 7).

### Quality of evidence assessment

To evaluate the level of evidence for our findings, we used the Grading of Recommendations, Assessment, Development, and Evaluations (GRADE) framework with the GRADEpro tool (22) (Detailed in Appendix 8).

## Results

### Study selection

**Figure 1:**
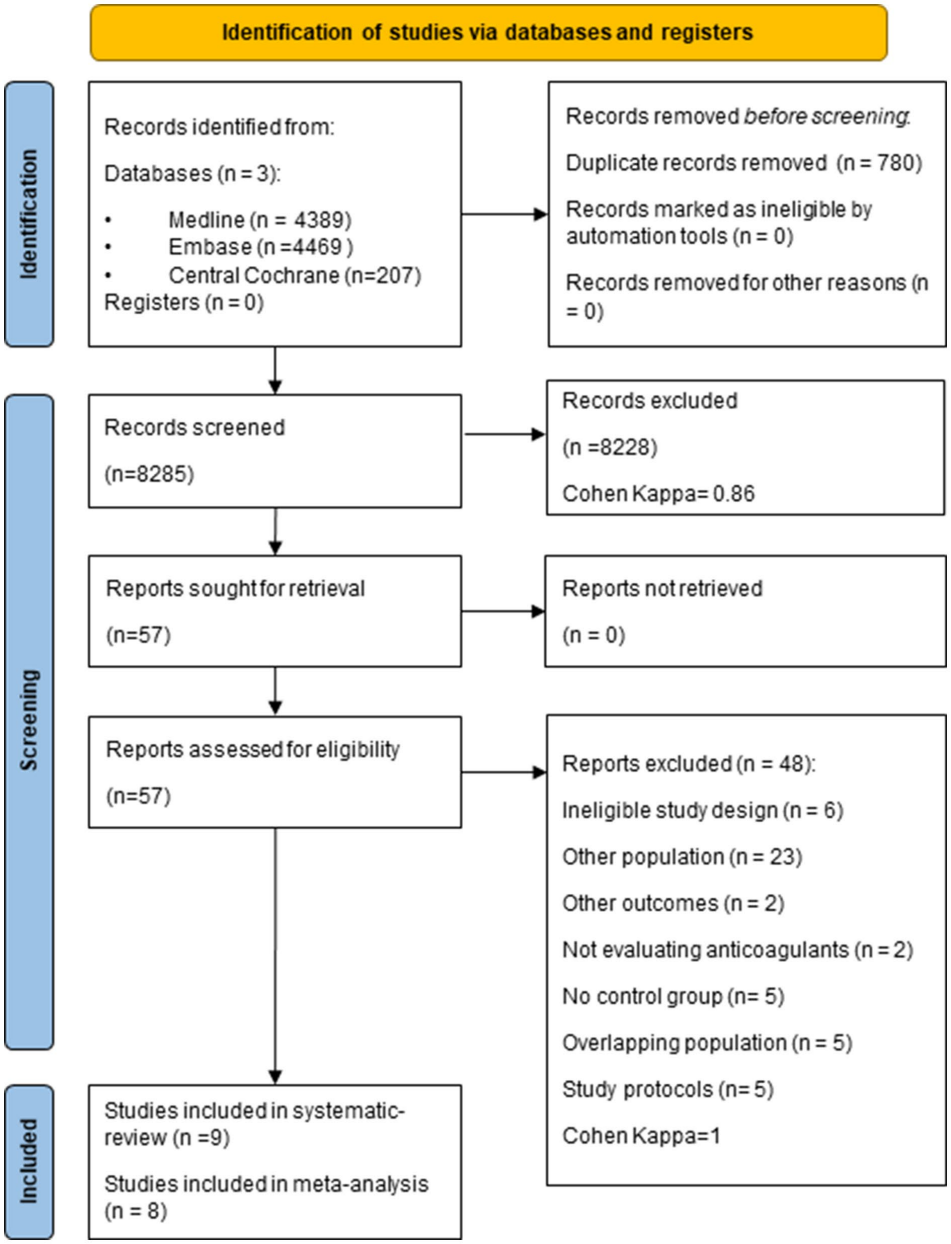
PRISMA flow chart describing the selection of studies for the systematic review and meta-analysis.

Our search strategy yielded 8,285 studies after removing duplicates, and after selection, 57 articles were eligible for full-text review. In total, nine studies fulfilled the eligibility criteria for the qualitative synthesis (8, 9, 23–29) and eight for the quantitative analysis. Five identified eligible reports were excluded due to overlapping populations (27, 30–33). The selection strategy is detailed in the PRISMA flow chart (Figure 1). No additional articles were found by screening the reference lists of the included papers.

### Main characteristics of the included studies

Of the nine eligible studies, five were RCTs (8, 9, 23, 24, 29), three were prospective cohort studies (25, 26, 28), and one was a retrospective propensity-matched study (27). The main characteristics of the included studies are summarized in Supplementary Table S1.

Duration of anticoagulant administration ranged from 1 to 14 days. In almost all studies, LMWH was used in the intervention group, except for the study of Kroner et al. (27) in which the types of anticoagulants were not stated. The dose of LMWH varied in the included studies as 1 mg/kg/per day, corresponding to the prophylactic dose or 2 mg/kg/per day, or 40 mg/per day corresponding to the therapeutic dose (34) respectively. Our pooled results reflect therefore mainly the effects of LMWH in patients with AP and we will report them accordingly.

### Quantitative synthesis

#### The mortality rate is decreased in moderate and severe acute pancreatitis patients receiving LMWH

**Figure 2:**
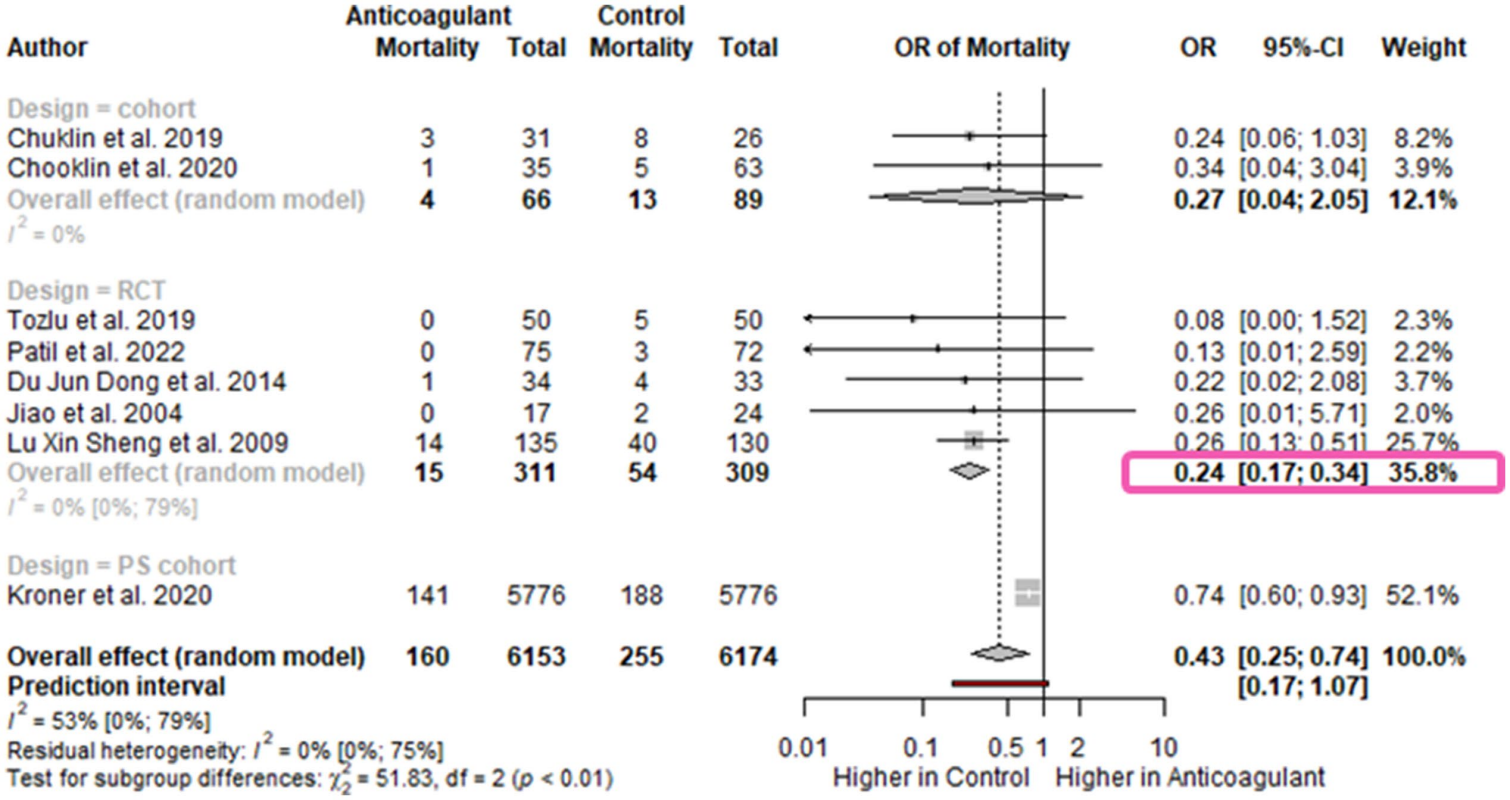
Forest plot for mortality**-**subgroup for study design. The mortality rate is decreased in AP patients receiving anticoagulants. OR, odds ratio; CI, confidence interval.

All studies included in the meta-analysis reported on the in-hospital mortality rate. In our analysis, 6,153 patients were included in the intervention group and 6,174 in the control group. The pooled OR was significantly lower in the LMWH group [OR 0.43; 95%CI 0.25–0.74, *I*^2^ = 53%; 95%CI 0–79%] (Figure 2). We performed subgroup analysis according to the study design. The RCTs yielded a similar pooled effect size [OR 0.24; 95%CI 0.17–0.34; *I*^2^ = 0%; 95%CI 0–79%], although based on a lower number of studies (Figure 2).

The subgroup analysis based on the disease severity resulted in similar results for moderately-severe [OR0.34; 95%CI 0.04–3.04] and severe AP [OR 0.25; 95%CI 0.22–0.29; *I*^2^=0%; 95%CI 0–90%]. This analysis included a limited number of studies–one in the moderately-severe subgroup (25), and three in the severe subgroup (Supplementary Figure S1) (8, 16, 23).

Moreover, we performed a subgroup analysis by the dose of LMWH. The odds for mortality was slightly lower for patients who received a therapeutic dose of LMWH [OR 0.16; 95%CI 0.07–0.36; *I*^2^ = 0%; 95%CI 0–85%] compared to those who received a prophylactic dose [OR 0.26; 95%CI 0.21–0.33; *I*^2^ = 0%; 95%CI 0–90%]. However, the differences between the groups were not significant according to Cochran’s Q test [*X*^2^ = 3.39; *p* = 0.07], though admittedly this test lacks statistical power when only a low number of studies is analyzed (Supplementary Figure S2).

Regarding the duration of therapy, no significant difference was detected with respect to the mortality rates for patients receiving shorter-duration LMWH treatment (ranging from 1 to 7 days) [OR 0.24; 95%CI 0.15–0.40; *I*^2^ = 0 95%CI 0–85%] versus longer-duration anticoagulation treatment (ranging from 12 to 14 days) [OR 0.26; 95%CI 0.16.0.42; *I*^2^ = 0 95%CI 0–85%], *X*^2^ = 0.12 *p* = 0.73 (Supplementary Figure S3).

### Low-molecular-weight heparin decrease the organ failure rate in acute pancreatitis

Five studies reported MOF rates - four RCTs (8, 9, 23, 29) and one propensity-matched cohort (27). LMWH treatment was associated with a lower rate of MOF but without statistical significance [OR 0.50; 95%CI 0.20–1.22; *I*^2^ = 65%; 95%CI 8–87%] (Supplementary Figure S4). The associated heterogeneity for this result was high. However, in the subgroup analysis for RCTs, the results were also in favor of LMWH treatment and reached statistical significance [OR 0.32; 95%CI 0.17–0.62; *I*^2^ = 0%; 95%CI 0–85%] (Supplementary Figure S4).

Additionally, we could analyze ARDS rates which was reported in 3 RCTs (8, 9, 29) and is significantly decreased in LMWH group [OR 0.28; 95%CI 0.16–0.48; *I*^2^=0%; 95%CI 0–90%] (Supplementary Figure S5).

We could also specifically evaluate the differences in AKI rates by pooling the results of three RCTs (8, 9, 29), and the propensity score-matched cohort (27). Our results showed there is no significant difference by adding LMWH with respect to AKI rates [OR 0.91; 95%CI 0.80–1.03; *I*^2^ = 0%; 95%CI 0–85%] (Supplementary Figure S6).

### Low-molecular-weight heparin decrease the need for endoscopic or surgical intervention in moderate and severe acute pancreatitis patients

**Figure 3:**
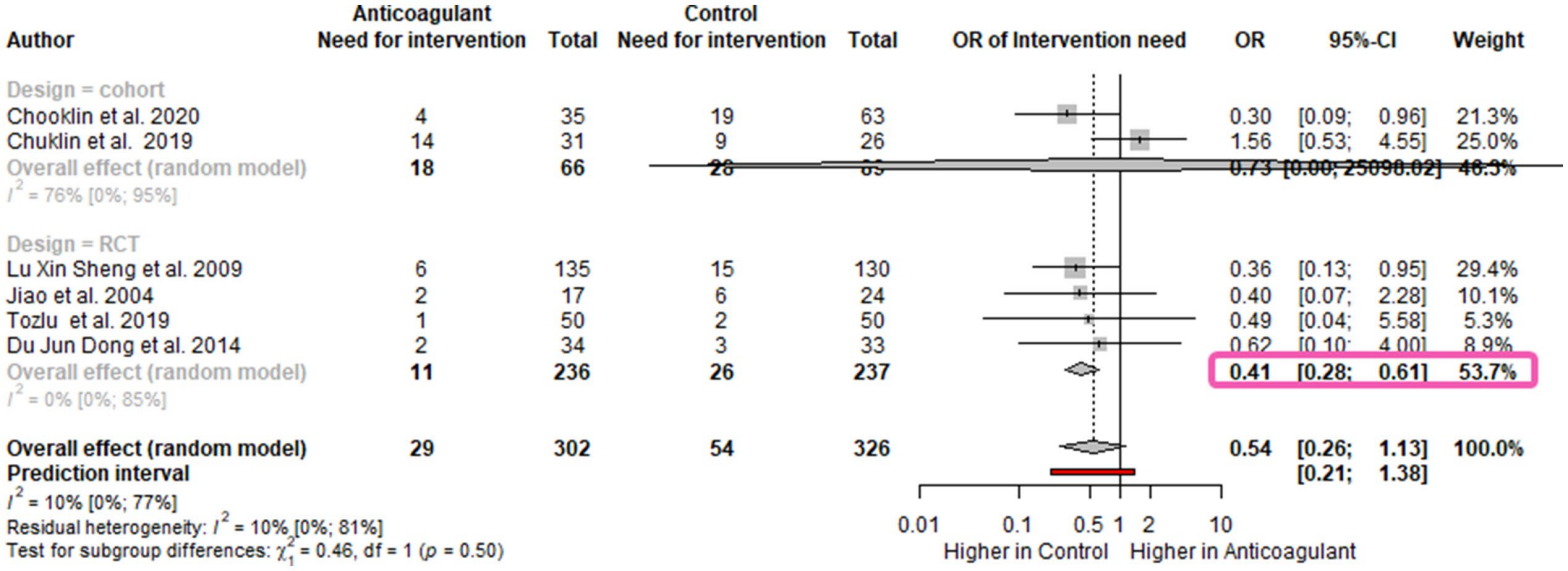
Forest plot for the need for endoscopic or surgical intervention-subgroup for study design Need for endoscopic or surgical intervention is decreased in AP patients. OR, odds ratio; CI, confidence interval.

The need for endoscopic or surgical interventions was assessed in six studies (8, 9, 23–26) and was also lower in the group receiving LMWH [OR 0.54; 95%CI 0.26–1.13; *I*^2^ = 10%; 95%CI 0–77%] (Figure 3). Here also, the RCTs subgroup analysis showed a significant difference between the groups in favor of LMWH [OR 0.41; 95%CI 0.28–0.61; *I*^2^=0%; 95%CI 0–85%] (Figure 3). Moreover, the subgroup analysis based on the disease severity revealed that the odds of having further interventions for moderately severe cases were decreased by 70% in the LMWH group [OR 0.30; 95%CI 0.09–0.96]. For the severe cases, the need for intervention was 30% lower in the LMWH group, yet without statistical significance [OR 0.70; 95%CI 0.09–5.30; *I*^2^=49%; 95%CI 0–85%] (Supplementary Figure S7). The type of intervention was not detailed across all the eligible articles.

### The length of hospital stay is 5 days shorter for AP patients receiving LMWH

**Figure 4:**
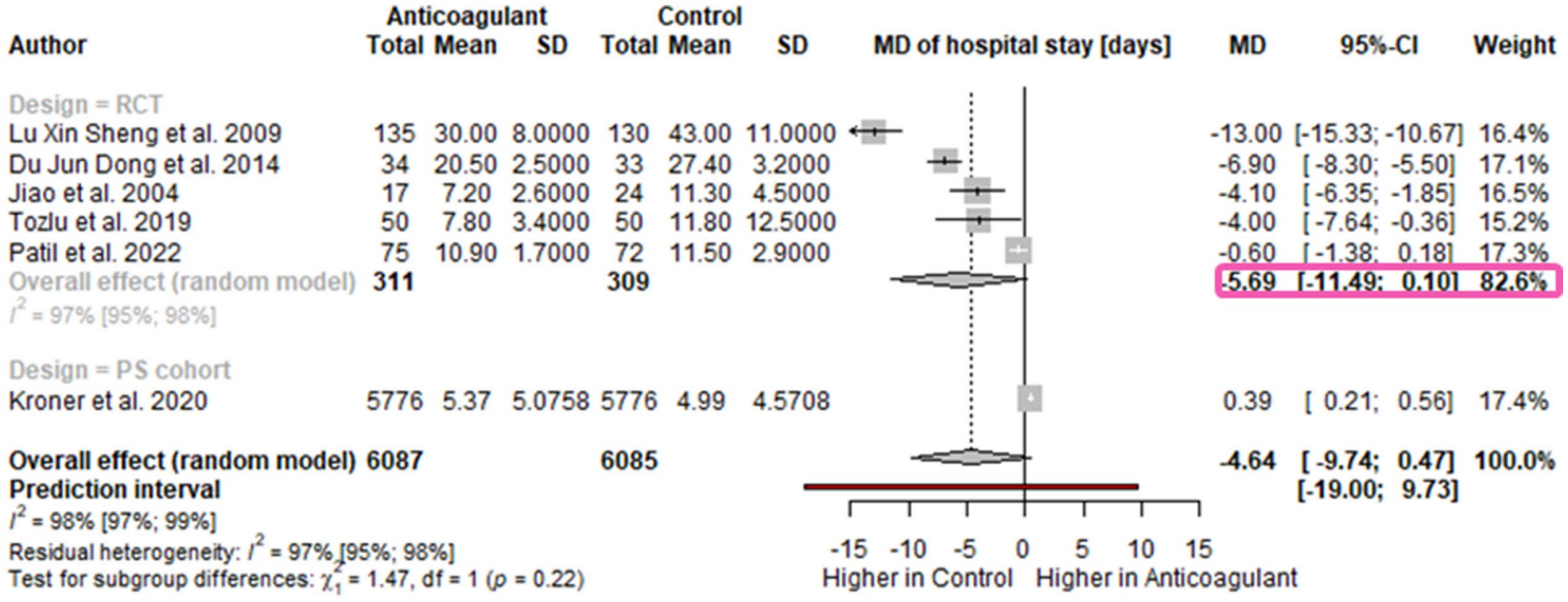
Forest plot for the length of hospital stay- subgroup for study design. Length of hospital stay is shorter in the anticoagulant group. MD, mean difference; CI, confidence interval.

Six studies reported the LOH (in days): five RCTs (8, 9, 23, 24, 29) and one propensity score-matched cohort (27). Our pooled results showed a reduction of admission duration of almost 5 days in AP patients receiving anticoagulants without statistical signification [MD-4.64 days; 95%CI -9.74-0.47; *I*^2^=96%; 95%CI 97–99%] (Figure 4). The RCTs analysis revealed that LOH could be decreased by around 6 days in the LMWH group [MD -5.69 days; 95%CI -11.49-0.10; *I*^2^=97%; 95%CI 95–98%] however, without statistical significance (Figure 4).

### Qualitative synthesis

Due to insufficient data, we could not perform meta-analytical calculations for the rates of bleeding, thrombosis, local complications, or for the effect of anticoagulants on AP severity progression (Detailed in Supplementary Table S2).

Vascular complications were reported in two RCTs (8, 29), and was considerably lower in the groups that received LMWH. Tozlu et al. (8) revealed a rate of vascular complications in the LMWH group of 2% while in the control group it reached 14%. Similarly, Patil et al. (29) reported a lower incidence of vascular thrombosis in the intervention group of 1.4% vs. control 12.9%.

Reduction of severity was assessed using the APACHE II score in three studies, one RCT (9) and two observational studies (25, 26). Across these three studies, 201 patients were in the LMWH group and 219 in the control group. The APACHE II score values significantly improved in the intervention groups after treatment compared to the controls (Supplementary Table S3).

The effect of LMWH on local complications development was reported in two of the identified studies, but with different results. Tozlu et al. (8) revealed a 20% decrease in local complication rates in the LMWH group, whereas Lu et al. (9) reported no difference between the two groups.

One RCT included in our systematic review (9) reported fewer cases of gastrointestinal bleeding in the LMWH group by comparison with the control group (3.7% vs. 4.6%).

Timing of LMWH initiation: only 5 out of 9 eligible studies reported the timing of the initiation of anticoagulants. Only in one RCT comprising of 134 patients with severe AP, the LMWH was started after admission, although the exact time was not specified. The rate of in-hospital mortality was 2.9% in the intervention group compared to 5% in the control group (*p* < 0.05) (23). In the other 4 studies, anticoagulants were initiated immediately at admission. The mortality rate in the intervention group varied as follows: 0 in 2 of the RCTs (141 of cases in total) (8, 24), in which the intervention consisted of LMWH, 2.4% in a propensity score matched cohort of 11,552 patients (27) I which type of anticoagulants was not mentioned and 10.4% in an RCT of 265 cases also testing LMWH (9). However, we must emphasize that in this last study only severe cases were included and the mortality rate in the control group was higher (30.6%) while in the other studies, it ranged between 3.3 and 10% (8, 9). Regarding the effect on organ failure, we were able to identify 3 main sub-groups: the multi-organ failure group, the ARDS group and the AKI group. Within the MOF group, we found five publications, where four were RCT and 1 was a propensity matched cohort study. Tozlu et al. (9) published an RCT study where they included 100 patients with moderately severe and severe AP diagnosis. They randomized the 100 patients into two different groups where on received standard care therapy, while the other received standard care therapy + LMWH sc. (1 mg/kg sc. twice a day between 1 and 7 days). They showed that the local (peripancreatic fluid collections and peripancreatic necrosis) and the systematic complications were significantly lower in the LMWH group (*p* < 0.05) with no hemorrhagic complications. In this RCT, pancreatic necrosis were present in one patient in the LMWH group compared with 2 patients in the standard care group. Lu Xin et al. in their RCT randomized 265 patients into conventional therapy group (CT, *n* = 130) and, LMWH group (*n* = 135). In the CT group, out of 130 patients, 6 had ARDS, 3 had AKI and 6 had MOFs while in the LT group, 11 patients had ARDS, 9 patients had AKI and 7 patients had MOFs. The results were statistically insignificant (*p* > 0.05).

The only study in which type of anticoagulants was not mentioned is that of Kroner et al. who explored the association of systemic anticoagulation before AP onset with in-patient outocomes through a retrospective propensitive score matched cohort analysis. Overall they were able to include in the study 5,776 pairs (matched for age, gender, race, household income, Charlson comorbidity score, hospital characteristics, pancreatic cancer class, obesity, tobacco use and during weekend admission) and patients on anticoagulants although having loinger hospital stay and increased care costs, tended to have better prognosis with lower rates of ICU admission organ failure development, and in-hospital mortality–therefore their results were similar to those of the studies assessing LMWH specifically.

In our meta-analysis based on the included studies, the LMWH treatment was associated with a lower rate of MOF but without statistically significance. The detailed data analysis from the articles are shown in the Supplementary Figure S4.

### Risk of bias assessment

Overall, the risk of bias across the eligible studies for our systematic review was moderate or serious, mainly due to bias in the selection of the reported results. The results of the risk of bias assessment for observational studies based on ROBINS-I are summarized in Supplementary Figure S8, and the risk of bias assessment for RCTs as based on RoB 2 are in Supplementary Table S4.

Publication bias could be assessed for all outcomes using funnel plots, which are available in Supplementary Figures S9–S13. Based on the plots, a risk for publication bias may arise, but the power of the analysis is low due to the limited data.

Also, influential analysis was performed for all outcomes and identified possible outliers (Supplementary Figures S14–S21).

### Quality of evidence assessment

Based on the GRADE assessment, the quality of evidence for every outcome was low or very low when analyzing RCTs and observational studies together, and moderate when performing meta-analysis only on the RCTs (Supplementary Table S5).

## Discussion

In this systematic review and meta-analysis, we summarized the current evidence on the efficacy and safety of anticoagulant treatment in AP. Currently, there is no consensus regarding the use of anticoagulants in AP regardless of its severity (5, 6, 35–38), yet it is often prescribed in clinical practice,considering that previous studies have demonstrated beseides anti-thrombotic also anti-inflammatory, and anti-protease properties of anticoagulants (39–41).

### Low-molecular-weight heparin treatment reduces mortality rates by half in acute pancreatitis

AP mortality rate strongly depends on disease severity and ranges from 3% in mild cases to 30% in severe ones (42). Mortality rate across all studies was lower in the LMWH group but with a moderate level of evidence. This was caused by the variability regarding the recruited patients across the eligible studies. Kroner et al. (27) included an older population compared to the other studies.

### Low-molecular-weight heparin potentially decreases complication rates in acute pancreatitis

The thrombotic complications were only scarcely reported across the identified studies. In contrast with the findings of Tozlu et al. (8) and Patil et al. (29) that reported a lower rate of vascular complications in the intervention group, Vadlamudi et al. (43) reported in his retrospective cohort analysis – which included 389 patients – no statistically significant correlation between the incidence of splenic vein thrombosis and the use of anticoagulant therapy. However, this study included both acute and chronic pancreatitis.

### The effect of prophylactic versus therapeutic LMWH dose in AP

Yuan et al. (44) published in 2009 the results of their RCT that revealed low-dose LMWH reduces the rate of complication, mortality, and LOH in AP, without increasing the incidence of hemorrhage complications. Qui et al. (10) based on their meta-analysis and systematic review of RCT trials reported shorter hospital stay [pooled mean difference (95% confidence interval; CI) -8.79 (−11.18, −6.40), *p* < 0.01], lower mortality [pooled risk ratio RR (95% CI) 0.33 (0.24–0.44), *p* < 0.01], lower incidences of multiple organ failure [pooled RR (95% CI) 0.34 (0.23–0.52), *p* < 0.01], pancreatic pseudocyst [pooled RR (95% CI) 0.49 (0.27–0.90), *p* = 0.02], and operation rate [pooled RR (95% CI) 0.39 (0.31–0.50), *p* < 0.01] in patients with severe acute pancreatitis (10). Sixteen randomized controlled trials with 1,625 patients were included in the final analysis, most studies were from China. Their conclusion was the LMWH could improve the prognosis of SAP, and has a potential role in reducing hospital stay, mortality, incidences of multiple organ failure, pancreatic pseudocyst, and operation rate.

We sought to evaluate our eligible studies’ best practice patterns related to anticoagulant doses. There were minor differences between therapeutic and prophylactic heparin doses regarding mortality rates, suggesting patients can benefit from prophylactic doses in daily use. More studies are needed to compare the efficacy of low dose vs. high dose treatment.

### Adverse events associated with LMWH therapy in acute pancreatitis

When considering anticoagulant therapy in AP, we must be aware of the associated bleeding risk, which is a rare but potentially lethal complication of severe AP (45). In a retrospective cohort study conducted at the Mayo Clinic that analyzed splanchnic vein thrombosis in AP (46), anticoagulant treatment was not associated with a higher rate of bleeding events and concluded that anticoagulants are safe for the management of AP. Also, the prospective cohort study conducted by Zemskov et al. (28) evaluated the use of enoxaparin for patients with severe AP. The analysis included 41 participants and reported no bleeding events or thrombocytopenia in the anticoagulant group.

The available data in the literature did not reveal an increased risk of bleeding in AP patients receiving anticoagulants. Nevertheless, this outcome is rarely reported in AP patients receiving prophylactic anticoagulants. Only the RCT of Lu et al. (9) assessed the incidence of gastrointestinal bleeding, which is reduced in the anticoagulant group. Therefore, despite some concerns about administering anticoagulants in AP that may require urgent surgical or endoscopic intervention, the available data suggests anticoagulants are safe in AP patients in terms of bleeding risk.

### Strengths and limitations

Our review has several strengths: it is based on robust methodology, and, to our knowledge it is the most comprehensive and up-to-date on the topic (consisting of more than 6,000 patients). The level of evidence for some of our results is high, since we could perform subgroup analysis for RCTs. Moreover, this is the first quantitative synthesis that assesses the effect of anticoagulants in moderate cases of AP.

There are several limitations to this study as well. The analysis included a low number of studies, especially for the moderately-severe subgroup. Many of our results have substantial heterogeneity (11) resulting from the age differences between the enrolled populations across the studies, variability in the distribution of disease severity, and study design differences. Secondly, the duration of therapy varied among the studies. Even though we included all types of anticoagulants in our search key, most clinical trials report only the use of LMWH treatment.

### Implications for research

First and foremost, there is a paucity of data in the literature regarding the use of anticoagulants in mild AP cases. We found no study that reports specifically on this subgroup. Further RCTs assessing this category of patients will be necessary. Another important implication for research is that studies should report the safety outcomes related to anticoagulants, such as bleeding or thrombocytopenia.

### Implications for clinical practice

Regarding implications for practice, adding anticoagulant treatment is beneficial for moderately severe and severe cases of AP and may be used earlier in the course of the disease.

## Conclusion

Anticoagulants decrease major complication rates in moderately severe and severe AP. Prophylactic and therapeutic doses of LMWH might have similar effects. Administration of anticoagulant therapy in moderately severe AP cases leads to lower complication rates than in severe cases. Further studies are necessary to assess the effects of anticoagulants in mild AP and to confirm their safety profile.

## Data availability statement

The original contributions presented in the study are included in the article/Supplementary material, further inquiries can be directed to the corresponding author.

## Author contributions

CP: conceptualization, project administration, investigation, formal analysis, writing–original draft, and visualization. DV: formal analysis, visualization, and writing–review and editing. SB: conceptualization, supervision, writing–review and editing, and methodology. DP: writing–review and editing. BE: conceptualization, supervision, and writing–review and editing. FD: methodology and writing–review and editing. LF: formal analysis and writing–review and editing. AÉ: investigation and writing–review and editing. PJH: conceptualization, methodology, supervision, and writing–original draft. PH: conceptualization, funding acquisition, and writing–review and editing. All authors certify that they have participated sufficiently in this work to take public responsibility for the content, including participation in the concept, design, analysis, writing, or revision of the manuscript. All authors contributed to the article and approved the submitted version.

## Abbreviations

AP: acute pancreatitis
AKI: Acute kidney injury
APACH: Acute Physiology and Chronic Health Evaluation
ARDS: Acute respiratory distress syndrome
CI: confidence interval
LOH: length of hospital stay
LMWH: low molecular weight heparin
MD: mean difference
MOF: multiple organ failure
OR: odds ratio
PICO: population-intervention-control-outcome
RCT: randomized control trial
RoB 2: risk-of-bias tool for randomized trials
ROBINS-I: Risk Of Bias In Non-randomized Studies - of Interventions
SOC: standard of care
