# Supplementary material for: Low molecular weight heparin decreases mortality and major complication rates in moderately severe and severe acute pancreatitis–a systematic review and meta-analysis
Source: Front Med (Lausanne). 2023 Oct 25;10:1241301. doi: 10.3389/fmed.2023.1241301 (PMC10630914; doi:10.3389/fmed.2023.1241301)
Supplement: Supplementary file 1 [file Data_Sheet_1.docx]

**Supplementary material**

**Anticoagulants decrease mortality and major complication rates in moderately severe and severe acute pancreatitis – a systematic review and meta-analysis**

**AUTHORS**

**Cristina Patoni**^1,2^, Stefania Bunduc^1,2,3,4^, Levente Frim^5^, Dániel Sándor Veres^1,6^, Fanni Dembrovszky ^1,5^, Anna Júlia Éliás^1,7^, Dániel Pálinkás^1,8^, Péter Hegyi^1,3,5,9^ **Bálint Erőss***^1,3,5^, **Péter Jenő Hegyi** *^1,3^

*equally contributed

**AFFILIATIONS**

^1^Center for Translational Medicine, Semmelweis University, Budapest, Hungary

^2^Carol Davila University of Medicine and Pharmacy, Bucharest, Romania

^3^Division of Pancreatic Diseases, Heart and Vascular Center, Semmelweis University, Budapest, Hungary

^4^Fundeni Clinical Institute, Bucharest, Romania

^5^Institute for Translational Medicine, Medical School, University of Pécs, Pécs, Hungary

^6^Department of Biophysics and Radiation Biology, Semmelweis University, Budapest, Hungary

^7^Doctoral School of Health Sciences, Semmelweis University, Budapest, Hungary

^8^ Department of Gastroenterology, Military Hospital – State Health Centre, Budapest, Hungary

^9^János Szentágothai Research Center, University of Pécs, Pécs, Hungary

**Email addresses**: [patonicristina@gmail.com](mailto:patonicristina@gmail.com) [levi.frim@gmail.com](mailto:levi.frim@gmail.com) [stfnbndc@gmail.com](mailto:stfnbndc@gmail.com) [daniel.s.veres@gmail.com](mailto:daniel.s.veres@gmail.com) [elias.anna.julia@gmail.com](mailto:elias.anna.julia@gmail.com) [dr.d.palinkas@gmail.com](mailto:dr.d.palinkas@gmail.com) [dr.eross.balint@gmail.com](mailto:dr.eross.balint@gmail.com) [hegyi2009@gmail.com](mailto:hegyi2009@gmail.com) [drdunajskastreda@gmail.com](mailto:drdunajskastreda@gmail.com)

**CORRESPONDING AUTHOR**

Name: Hegyi Péter Jenő

Email: [drdunajskastreda@gmail.com](mailto:drdunajskastreda@gmail.com)

Fax, phone number: +421918259560

**TABLE OF CONTENTS**

Supplementary Appendix 1-4. Methods

Supplementary Table 1. PRISMA checklist

Supplementary Table 2. Outcomes available for qualitative synthesis

Supplementary Table 3. APACHE II score

Supplementary Table 4. Risk of bias for randomized clinical trials

Supplementary Table 5. GRADE Working Group grades of evidence

Supplementary Figure 1-7. Supplementary forest plots

Supplementary Figure 8. Summary plot for risk of bias assessment with Robins-I

Supplementary Figure 9-13. Assessment of publication bias

Supplementary Figure 14-21. Sensitivity analysis

References

**Methods**

**Appendix 1**

We applied the following search key in all three databases: *(anticoagula* OR anticoagulants OR DOAC OR NOAC OR "non-vitamin K antagonist" OR "vitamin K antagonist" OR coumarins OR warfarin OR acenocoumarol OR phenprocoumon OR atromentin OR phenindione OR heparin OR "low molecular weight heparin" OR LMWH OR ULMWH OR fondaparinux OR enoxaparin OR lovenox OR dalteparin OR idraparinux OR idrabiotaparinux OR dabigatran OR rivaroxaban OR apixaban OR edoxaban OR betrixaban OR edoxaban OR betrixaban OR hirudin OR lepirudin OR bivalirudin OR argatroban)* ***AND*** *pancreatitis*.

We manually screened the reference lists of the included studies for additional eligible articles.

**Appendix 2**

Cohen's Kappa coefficient (k) was calculated to evaluate inter-rater agreement during each selection step, with the following significance: k ≤ 0 - no agreement; k=0.10-0.20 - none or slight agreement; k = 0.21-0.4 - fair agreement; k = 0.41-0.60 - moderate agreement; k = 0.61-0.80 - substantial agreement; k = 0.81-1.00 - almost perfect agreement.

In cases of multiple reports from a single research group on the same or overlapping population, data from the most recent and/or most comprehensive report were retained.

**Appendix 3**

The following data was extracted into a standardized Excel (Microsoft Corporation, Redmond, Washington, USA) datasheet publication details - authors, year of publication, country of origin, Digital Object Identifier (DOI); study characteristics - study type, follow-up period, sample size; patient characteristics: age, sex distribution, disease severity classified as mild, moderate or severe, comorbidities; intervention data: type of anticoagulant, dose, and route of administration; and outcomes: primary outcome: in-hospital mortality rate; secondary outcomes: LOH, complications rates - local and systemic (MOF, AKI, pleural effusion, ARDS, pancreatic necrosis, pseudocysts), the need for surgery or endoscopic interventions, progression of severity, laboratory parameters, thrombotic events, and bleeding events as reported in each study.

**Appendix 4**

Pooled OR was calculated by the Mantel-Haenszel method.^1-3^ Exact Mantel-Haenszel method (without continuity correction) was used to handle zero cell counts. ^4^ ^5^ Inverse variance weighting method was used to calculate the pooled mean difference.

**Appendix 5**

We assumed that all subgroups share a common τ^2^ value as we did not anticipate difference in the between-study heterogeneity in the subgroups and the study number is relatively small in some subgroup. To assess the difference between the subgroups a Cochrane Q test was used between subgroups.^6^ The null hypothesis was rejected on a 5% significance level.

**Appendix 6**

Outlier and influence analyses were carried out following the recommendations of Harrer et al.^6^ and Viechtbauer and Cheung.^7^ Publication bias was assessed with Egger’s test (at significance level of 10% as a small study number) using the Harbord method for binary outcome measures, ^8^ and classical Egger’s method for continuous outcomes to calculate the test statistic.^9^ The analysis results was critically handled while the study number was below 10, and the study effects showed high heterogeneity.

**Appendix 7**

ROB 2 evaluated five main domains: randomization process, deviations from intended interventions, missing outcome data, measurement of the outcome, and selection of the reported results.

The evaluated domains for ROBINS-I were: confounding and selection of participants into the study, classification of interventions, deviations from intended interventions, missing data, measurement of outcomes, and selection of the reported result.

**Appendix 8**

Each outcome was rated for risk of bias, inconsistency, indirectness, imprecision, publication bias, the presence of a large effect, dose-dependent response, and plausible confounders as 'not serious', 'serious', or 'very serious'. The final certainty of the evidence was categorized as 'very low', 'low', 'moderate', or 'high'.

**Supplementary Table 1.** PRISMA checklist

| **Section and TtaTopic** | **Item #** | **Checklist itm** | **Location where item is reported** |
| --- | --- | --- | --- |
| **TITLE** | | |  |
| Title | 1 | Identify the report as a systematic review. | 1 |
| **ABSTRACT** | | |  |
| Abstract | 2 | See the PRISMA 2020 for Abstracts checklist. | 4-5 |
| **INTRODUCTION** | | |  |
| Rationale | 3 | Describe the rationale for the review in the context of existing knowledge. | 6 |
| Objectives | 4 | Provide an explicit statement of the objective(s) or question(s) the review addresses. | 6 |
| **METHODS** | | |  |
| Eligibility criteria | 5 | Specify the inclusion and exclusion criteria for the review and how studies were grouped for the syntheses. | 7-8 |
| Information sources | 6 | Specify all databases, registers, websites, organisations, reference lists and other sources searched or consulted to identify studies. Specify the date when each source was last searched or consulted. | 7 |
| Search strategy | 7 | Present the full search strategies for all databases, registers and websites, including any filters and limits used. | 8  +Supplementary material Appendix 1 |
| Selection process | 8 | Specify the methods used to decide whether a study met the inclusion criteria of the review, including how many reviewers screened each record and each report retrieved, whether they worked independently, and if applicable, details of automation tools used in the process. | 8 |
| Data collection process | 9 | Specify the methods used to collect data from reports, including how many reviewers collected data from each report, whether they worked independently, any processes for obtaining or confirming data from study investigators, and if applicable, details of automation tools used in the process. | 8-9  +Supplementary material Appendix 2 |
| Data items | 10a | List and define all outcomes for which data were sought. Specify whether all results that were compatible with each outcome domain in each study were sought (e.g. for all measures, time points, analyses), and if not, the methods used to decide which results to collect. | 8-9 |
|  | 10b | List and define all other variables for which data were sought (e.g. participant and intervention characteristics, funding sources). Describe any assumptions made about any missing or unclear information. | 8-9 |
| Study risk of bias assessment | 11 | Specify the methods used to assess risk of bias in the included studies, including details of the tool(s) used, how many reviewers assessed each study and whether they worked independently, and if applicable, details of automation tools used in the process. | 10  +Supplementary material Appendix 6 |
| Effect measures | 12 | Specify for each outcome the effect measure(s) (e.g. risk ratio, mean difference) used in the synthesis or presentation of results. | 9 |
| Synthesis methods | 13a | Describe the processes used to decide which studies were eligible for each synthesis (e.g. tabulating the study intervention characteristics and comparing against the planned groups for each synthesis (item #5)). | 9 |
|  | 13b | Describe any methods required to prepare the data for presentation or synthesis, such as handling of missing summary statistics, or data conversions. | 8-9 |
|  | 13c | Describe any methods used to tabulate or visually display results of individual studies and syntheses. | 9 |
|  | 13d | Describe any methods used to synthesize results and provide a rationale for the choice(s). If meta-analysis was performed, describe the model(s), method(s) to identify the presence and extent of statistical heterogeneity, and software package(s) used. | 9  +Supplemntary material Appendix 3 and 4 |
|  | 13e | Describe any methods used to explore possible causes of heterogeneity among study results (e.g. subgroup analysis, meta-regression). | 9 |
|  | 13f | Describe any sensitivity analyses conducted to assess robustness of the synthesized results. | 9  +Supplementary material- Appendix 5 |
| Reporting bias assessment | 14 | Describe any methods used to assess risk of bias due to missing results in a synthesis (arising from reporting biases). | 10 |
| Certainty assessment | 15 | Describe any methods used to assess certainty (or confidence) in the body of evidence for an outcome. | 10 |
| **RESULTS** | | |  |
| Study selection | 16a | Describe the results of the search and selection process, from the number of records identified in the search to the number of studies included in the review, ideally using a flow diagram. | 10  +Figure 1 |
|  | 16b | Cite studies that might appear to meet the inclusion criteria, but which were excluded, and explain why they were excluded. | 10 |
| Study characteristics | 17 | Cite each included study and present its characteristics. | 11  +Table 1 |
| Risk of bias in studies | 18 | Present assessments of risk of bias for each included study. | 15 + Supplementary Figure 7 and Supplementary Table 4 |
| Results of individual studies | 19 | For all outcomes, present, for each study: (a) summary statistics for each group (where appropriate) and (b) an effect estimate, and its precision (e.g. confidence/credible interval), ideally using structured tables or plots. | 11-15  +Figures 2-4  +Supplementary Figures 1-10  +Supplementary Tables 2-3 |
| Results of syntheses | 20a | For each synthesis, briefly summarise the characteristics and risk of bias among contributing studies. | Supplementary Figure 11 and Supplementary Table 4 |
|  | 20b | Present results of all statistical syntheses conducted. If meta-analysis was done, present for each the summary estimate and its precision (e.g. confidence/credible interval) and measures of statistical heterogeneity. If comparing groups, describe the direction of the effect. | 11-15  +Figures 2-4  +Supplementary Figures 1-10  +Supplementary Tables 2-3 |
|  | 20c | Present results of all investigations of possible causes of heterogeneity among study results. | 15  +Supplementary figures 17-24 |
|  | 20d | Present results of all sensitivity analyses conducted to assess the robustness of the synthesized results. | 15  +Supplementary material |
| Reporting biases | 21 | Present assessments of risk of bias due to missing results (arising from reporting biases) for each synthesis assessed. | 15  +Supplementary material |
| Certainty of evidence | 22 | Present assessments of certainty (or confidence) in the body of evidence for each outcome assessed. | 15  +Supplementary Table 5 |
| **DISCUSSION** | | |  |
| Discussion | 23a | Provide a general interpretation of the results in the context of other evidence. | 16-19 |
|  | 23b | Discuss any limitations of the evidence included in the review. | 19 |
|  | 23c | Discuss any limitations of the review processes used. | 19 |
|  | 23d | Discuss implications of the results for practice, policy, and future research. | 20 |
| **OTHER INFORMATION** | | |  |
| Registration and protocol | 24a | Provide registration information for the review, including register name and registration number, or state that the review was not registered. | 7 |
|  | 24b | Indicate where the review protocol can be accessed, or state that a protocol was not prepared. | 7 |
|  | 24c | Describe and explain any amendments to information provided at registration or in the protocol. | Not applicable |
| Support | 25 | Describe sources of financial or non-financial support for the review, and the role of the funders or sponsors in the review. | 21 |
| Competing interests | 26 | Declare any competing interests of review authors. | 21 |
| Availability of data, code and other materials | 27 | Report which of the following are publicly available and where they can be found: template data collection forms; data extracted from included studies; data used for all analyses; analytic code; any other materials used in the review. | 22 |

**Supplementary Table 2. Outcomes available for qualitative synthesis**

| Study's first author and year of publication | Outcome | Total number of patients anticoagulant group | Number of events anticoagulant group | Total number of patients control group | Number of events control group |
| --- | --- | --- | --- | --- | --- |
| Tozlu et al. 2019 | Vascular complication | 50 | 1 | 50 | 7 |
| Patil et al. 2022 | Vascular thrombosis | 75 | 1 | 72 | 9 |
| Lu Xin Sheng et al. 2009 | Diffuse intravascular clotting | 135 | 0 | 130 | 0 |
| Lu Xin Sheng et al. 2009 | Gastrointestinal bleeding | 135 | 5 | 130 | 6 |
| Tozlu et al. 2019 | Pleural effusion | 50 | 17 | 50 | 33 |
| Lu Xin Sheng et al. 2009 | Pancreatic encephalopathy | 135 | 3 | 130 | 13 |
| Kroner et al. 2020 | Need for ICU admission | 5776 | 307 | 5776 | 398 |
| Tozlu et al. 2019 | Local complications | 50 | 9 | 50 | 28 |

**ARDS:** Acute respiratory distress syndrome, **ICU**: intensive care unit

**Supplementary Table 3. Severity reduction in the anticoagulant vs control group**

| Study's first author and year of publication | Total number of patients (intervention/control) | Anticoagulant group | | Control group | |  |
| --- | --- | --- | --- | --- | --- | --- |
|  |  | APACHE II score on admission  (mean± SD) | APACHE II score after treatment  (mean± SD) | APACHE II score on admission  (mean± SD) | APACHE II score after treatment  (mean± SD) | *p* value |
| Chooklin et al. 2020 | 35/63 | 9.54 ± 1.01 | 5.52 ±1.31 | 9.48 ±1.01 | 7.49 ±1.58 | <0.05 |
| Chuklin et al 2019 | 31/26 | 11.94 ±1.39 | 6.96 ±1.63 | 12.42 ±1.42 | 10.18 ±1.44 | <0.05 |
| Lu Xin Sheng et al. 2009 | 135/130 | 11.6 ±3.6 | 8.5 ±1.8 | 11.5 ±3.4 | 9.6 ±2.4 | <0.05 |

**APACHE**: Acute Physiology and Chronic Health Evaluation; **SD**: standard deviation

**Supplementary Table 4.** Risk of bias for randomized clinical trials

| Study | Randomization process | Deviations from intended interventions | Missing outcome data | Measurement of the outcome | Selection of the reported result | Overall |  |
| --- | --- | --- | --- | --- | --- | --- | --- |
| MORTALITY | | | | | | |  |
| Du et al. | \|  \| \| --- \| | \|  \| \| --- \| | \|  \| \| --- \| | \|  \| \| --- \| | \|  \| \| --- \| | \|  \| \| --- \| |  |
| Lu et al. | \|  \| \| --- \| |  |  |  |  |  |  |
| Tozlu et al. | \|  \| \| --- \| |  |  |  |  |  |  |
| Jiao et al. | \|  \| \| --- \| | \|  \| \| --- \| | \|  \| \| --- \| | \|  \| \| --- \| | \|  \| \| --- \| | \|  \| \| --- \| |  |
| Patil et al. |  |  |  |  |  |  |  |
| MULTIPLE ORGAN FAILURE | | | | | | |  |
| Tozlu et al. | \|  \| \| --- \| | \|  \| \| --- \| | \|  \| \| --- \| | \|  \| \| --- \| | \|  \| \| --- \| | \|  \| \| --- \| |  |
| Lu et al. | \|  \| \| --- \| |  |  |  |  |  |  |
| Du et al. | \|  \| \| --- \| |  |  |  |  |  |  |
| Patil et al. |  |  |  |  |  |  |  |
| ACUTE KIDNEY INJURY | | | | | | |  |
| Tozlu et al | \|  \| \| --- \| | \|  \| \| --- \| | \|  \| \| --- \| | \|  \| \| --- \| | \|  \| \| --- \| | \|  \| \| --- \| |  |
| Lu et al | \|  \| \| --- \| |  |  |  |  |  |  |
| LENGTH OF HOSPITAL STAY | | | | | | |  |
| Tozlu et al | \|  \| \| --- \| | \|  \| \| --- \| | \|  \| \| --- \| | \|  \| \| --- \| | \|  \| \| --- \| | \|  \| \| --- \| |  |
| Lu et al | \|  \| \| --- \| |  |  |  |  |  |  |
| Du et al | \|  \| \| --- \| |  |  |  |  |  |  |
| Jiao et al | \|  \| \| --- \| |  |  |  |  |  |  |
| NEED FOR ENDOSCOPIC OR SURGICAL INTERVENTIONS | | | | | | |  |
| Lu et al | \|  \| \| --- \| | \|  \| \| --- \| | \|  \| \| --- \| | \|  \| \| --- \| | \|  \| \| --- \| | \|  \| \| --- \| |  |
| Du et al | \|  \| \| --- \| |  |  |  |  |  |  |
| Tozlu et al | \|  \| \| --- \| |  |  |  |  |  |  |
| Jiao et al | \|  \| \| --- \| | \|  \| \| --- \| | \|  \| \| --- \| | \|  \| \| --- \| | \|  \| \| --- \| | \|  \| \| --- \| |  |
| ACUTE RESPIRATORY DISTRESS SYNDROME | | | | | | |  |
| Lu et al. | | | | | | |  |
| Tozlu et al. | | | | | | |  |
| Patil et al. | | | | | | |  |

Low risk Some concerns High risk

**Supplementary Table 5**. GRADE Working Group grades of evidence

| **Certainty assessment** | | | | | | | **№ of patients** | | **Effect** | | **Certainty** | **Importance** |
| --- | --- | --- | --- | --- | --- | --- | --- | --- | --- | --- | --- | --- |
| **№ of studies** | **Study design** | **Risk of bias** | **Inconsistency** | **Indirectness** | **Imprecision** | **Other considerations** | **administration of anticoagulants** | **no anticoagulants** | **Relative (95% CI)** | **Absolute (95% CI)** |  |  |
| **Mortality** | | | | | | | | | | | | |
| 2 | observational studies | very serious | not serious | not serious | serious^a^ | dose response gradient | 4/66 (6.1%) | 13/89 (14.6%) | **OR --** (0.08 to 0.90) | **-- per 1.000** (from 133 fewer to 13 fewer) | ⨁⨁◯◯ Low | CRITICAL |
| **Mortality** | | | | | | | | | | | | |
| 5 | randomised trials | serious | not serious | not serious | not serious | none | 15/311 (4.8%) | 54/309 (17.5%) | **OR --** (0.17 to 0.34) | **-- per 1.000** (from 140 fewer to 108 fewer) | ⨁⨁⨁◯ Moderate | CRITICAL |
| **Multiple organ failure** | | | | | | | | | | | | |
| 4 | randomised trials | serious | not serious | not serious | not serious | none | 15/294 (5.1%) | 43/285 (15.1%) | **OR --** (0.17 to 0.62) | **-- per 1.000** (from 122 fewer to 52 fewer) | ⨁⨁⨁◯ Moderate | CRITICAL |
| **Acute kidney injury** | | | | | | | | | | | | |
| 2 | randomised trials | serious | not serious | not serious | very serious | none | 3/185 (1.6%) | 8/180 (4.4%) | **OR --** (0.1 to 1.4) | **-- per 1.000** (from 40 fewer to 17 more) | ⨁◯◯◯ Very low | IMPORTANT |
| **Need for further intervention** | | | | | | | | | | | | |
| 4 | randomised trials | not serious | not serious | not serious | serious^a^ | none | 11/236 (4.7%) | 26/237 (11.0%) | **OR --** (0.30 to 0.94) | **-- per 1.000** (from 74 fewer to 6 fewer) | ⨁⨁⨁◯ Moderate | IMPORTANT |
| **Need for further intervention** | | | | | | | | | | | | |
| 2 | observational studies | serious | serious^b^ | not serious | serious^a^ | none | 18/66 (27.3%) | 28/89 (31.5%) | **OR --** (0.32 to 1.68) | **-- per 1.000** (from 187 fewer to 121 more) | ⨁◯◯◯ Very low | IMPORTANT |
| **Length of hospital stay** | | | | | | | | | | | | |
| 5 | randomised trials | serious | not serious | not serious | not serious | none | 311 | 309 | - | **0**  (0 to 0 ) | ⨁⨁⨁◯ Moderate | IMPORTANT |
| **Acute respiratory distress syndrome** | | | | | | | | | | | | |
| 3 | randomised trials | serious | not serious | not serious | not serious |  | 20/260 (7.7%) | 55/252 (21.8%) | **OR --** (0.16 to 0.48) | **-- per 1.000** (from 175 fewer to 100 fewer) | - | IMPORTANT |

**CI:** confidence interval; **OR:** odds ratio **a**. Small sample size **b**. Studies show different results

**High certainty**: We are very confident that the true effect lies close to that of the estimate of the effect

**Moderate certainty**: We are moderately confident in the effect estimate: The true effect is likely to be close to the estimate of the effect, but there is a possibility

that it is substantially different

**Low certainty**: Our confidence in the effect estimate is limited: The true effect may be substantially different from the estimate of the effect

**Very low certainty**: We have very little confidence in the effect estimate: The true effect is likely to be substantially different from the estimate of effect


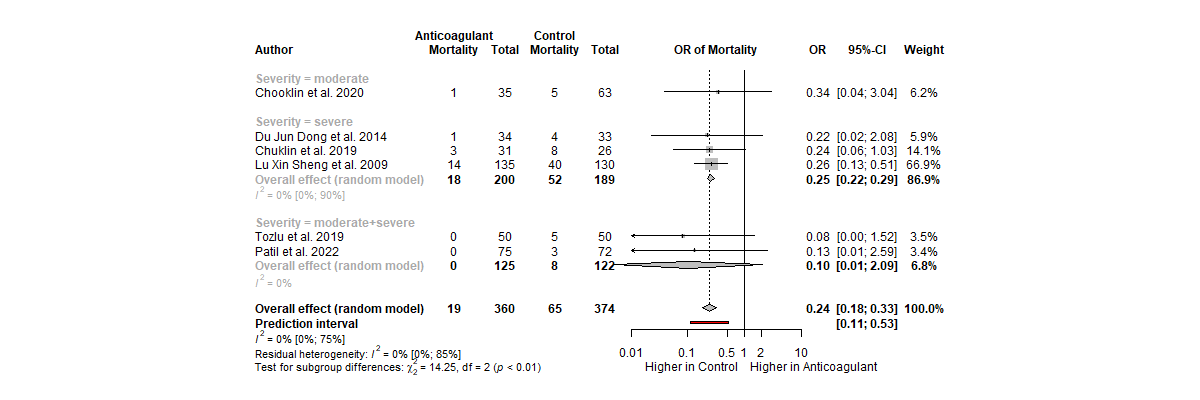


**Supplementary Figure 1.** Mortality – subgroup for severity: Patients with severe cases, benefit more in comparison with moderately severe cases. **OR:** odds ratio; **95%-CI**: 95% confidence interval


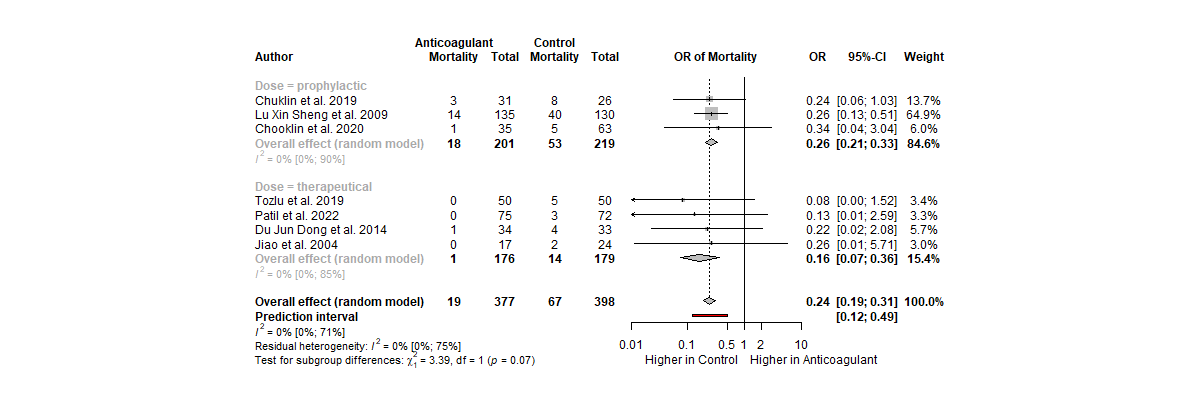


**Supplementary Figure 2.** Mortality- subgroup for dose**:** Patients may benefit more from receiving the therapeutical dose. **OR:** odds ratio; **95%-CI**: 95% confidence interval


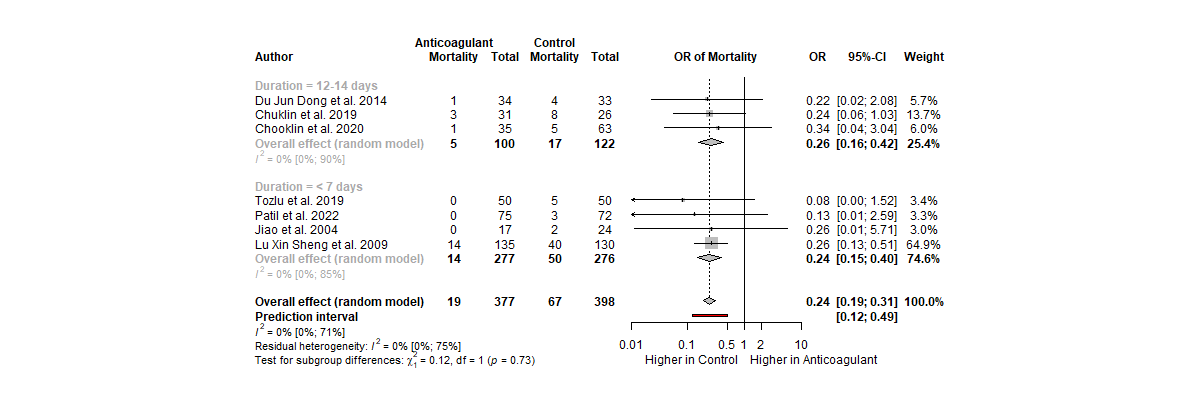


**Supplementary Figure 3.** Mortality- subgroup for duration of treatment **OR:** odds ratio; **95%-CI**: 95% confidence interval


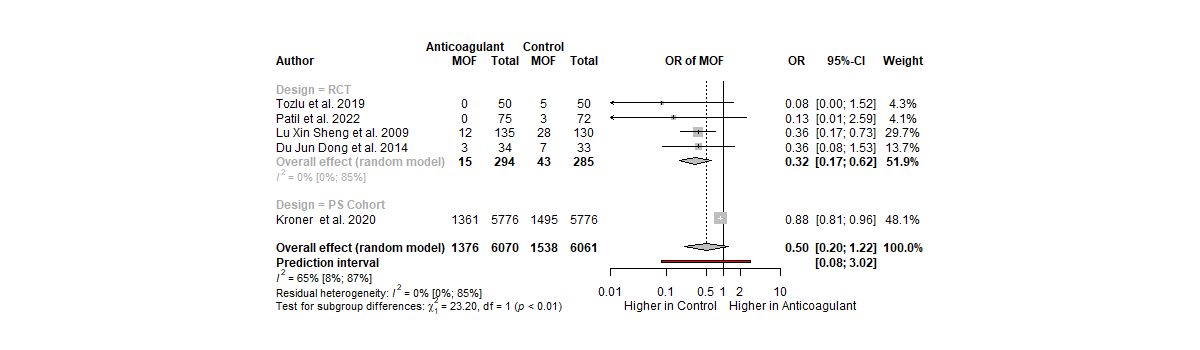


**Supplementary Figure 4.** Multiple organ failure- subgroup for design: Incidence of MOF decreased with 68% by adding anticoagulants. **OR:** odds ratio; **95%-CI**: 95% confidence interval


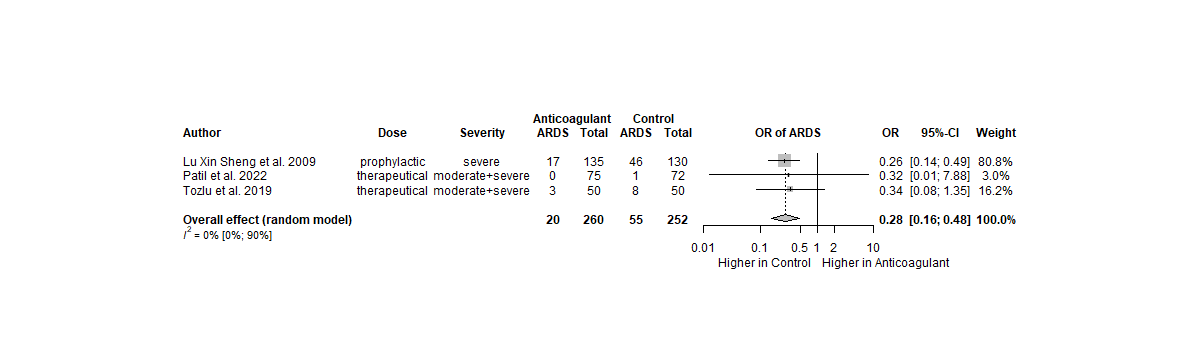


**Supplementary Figure 5.** Anticoagulants significantly decrease acute respiratory distress syndrome **OR:** odds ratio; **95%-CI**: 95% confidence interval


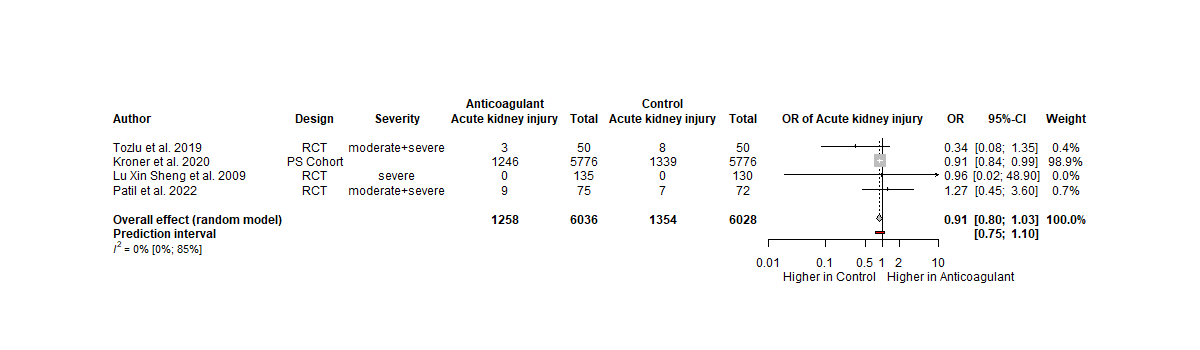


**Supplementary Figure 6.** Acute kidney injury: Anticoagulants are associated with lower rate of acute kidney injury in around 10 % of cases. **OR:** odds ratio; **95%-CI**: 95% confidence interval


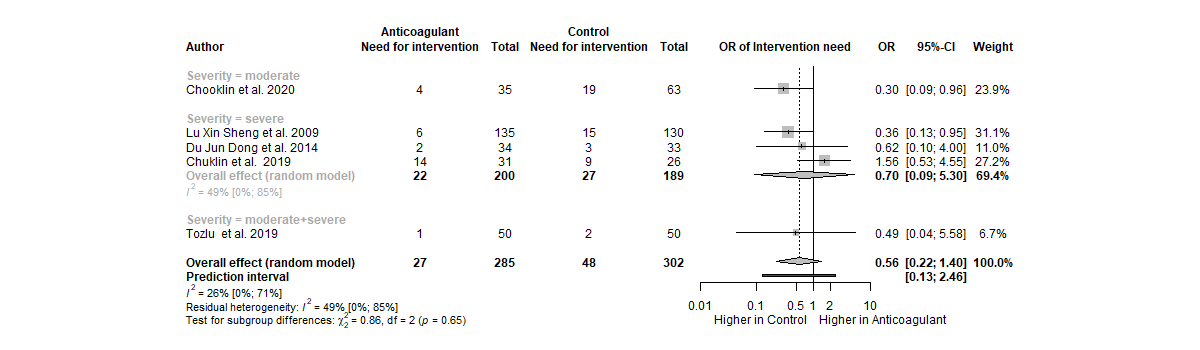


**Supplementary Figure 7.** Need for endoscopic or surgical intervention- subgroup for severity: Initiation of treatment early in the course of the disease has favorable results compared with late treatment. **OR:** odds ratio; **95%-CI**: 95% confidence interval

**
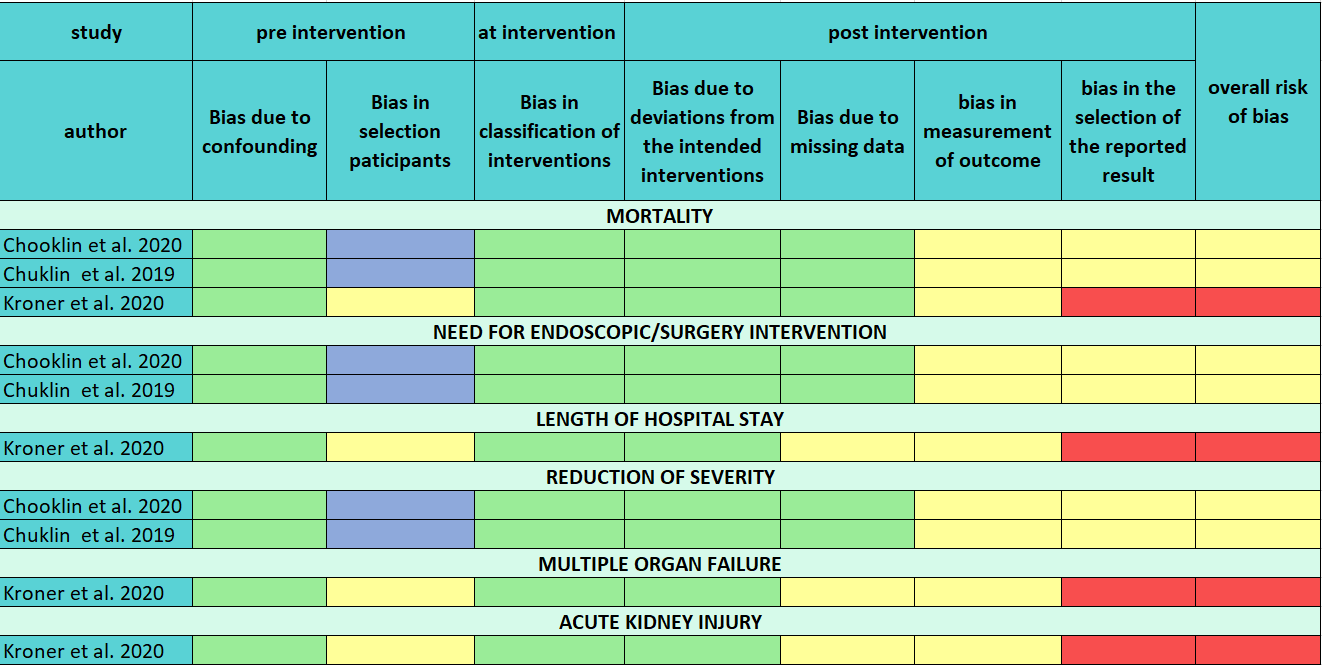
**

**Supplementary Figure 8.** Risk of bias for observational studies- ROBINS I


**Supplementary Figure 9.** Publication bias - Funnel plot for mortality

**Supplementary Figure 10.** Publication bias - Funnel plot for multiple organ failure

**Supplementary Figure 11.** Publication bias - Funnel plot for acute kidney injury

**Supplementary Figure 12.** Publication bias - Funnel plot for need for endoscopic or surgical interventions

**Supplementary Figure 13.** Publication bias - Funnel plot for length of hospital stay


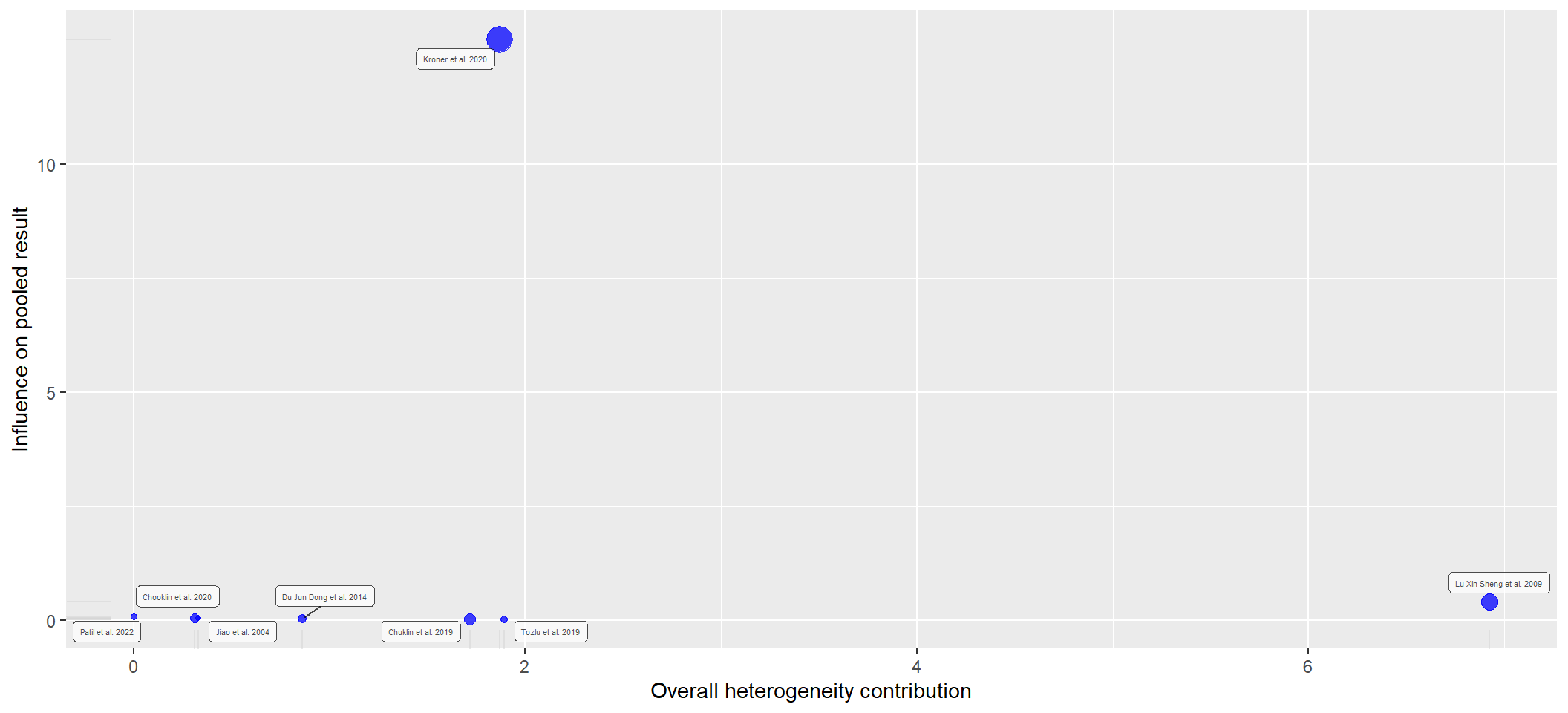


**Supplementary Figure 14**. Sensitivity analysis for mortality - Baujat plot: Two studies are potential outliers: Kroner et. al has a high influence on the pooled effect and Lu et al has a high impact on the heterogeneity.


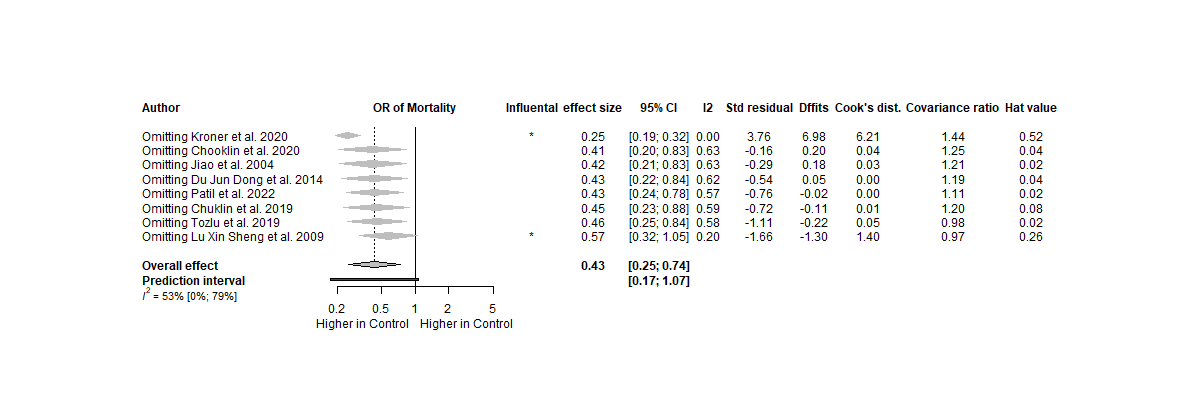


**Supplementary Figure 15**. Sensitivity analysis for mortality- Leave-one-out analysis: Kroner et. al and Lu et. al are potential outliers

**
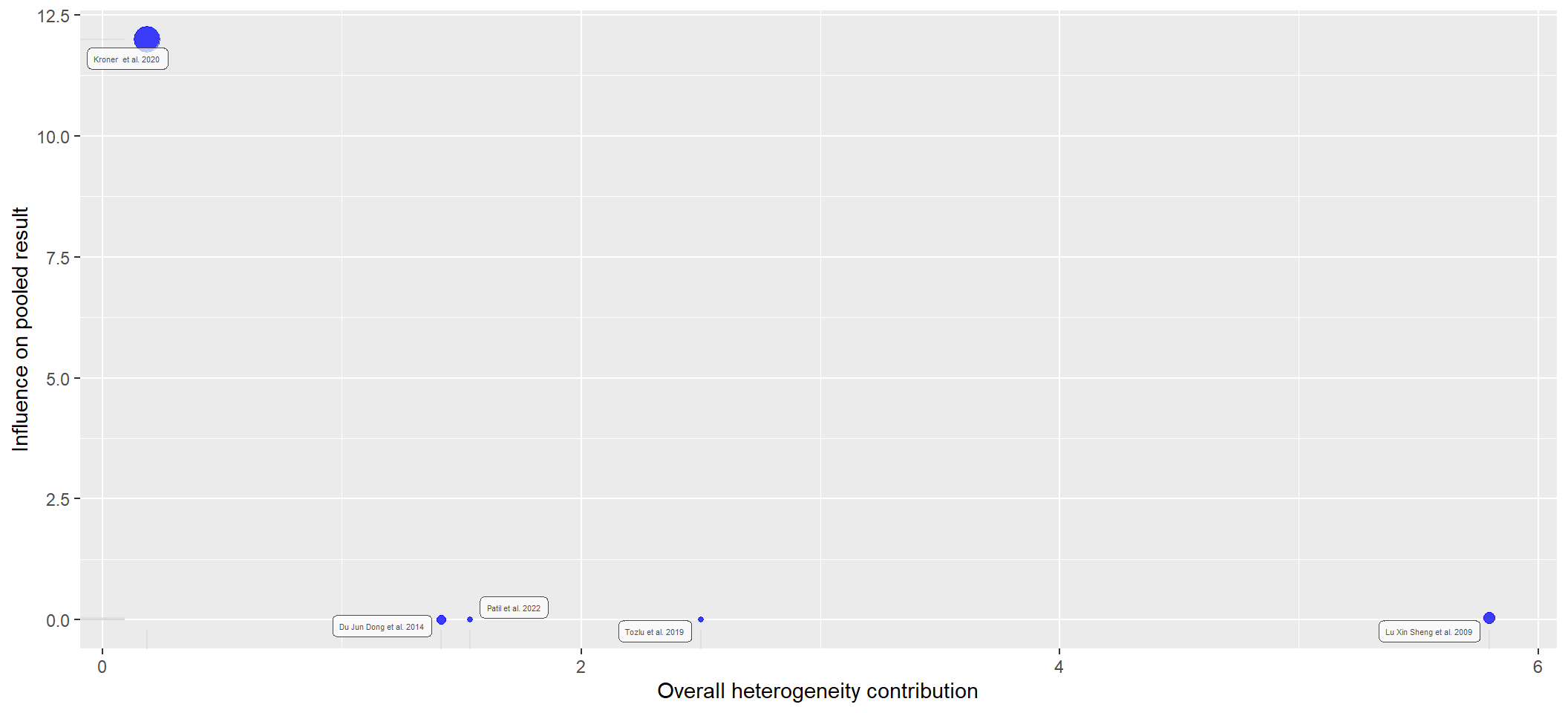
**

**Supplementary Figure 16**. Sensitivity analysis for multiple organ failure - Baujat plot


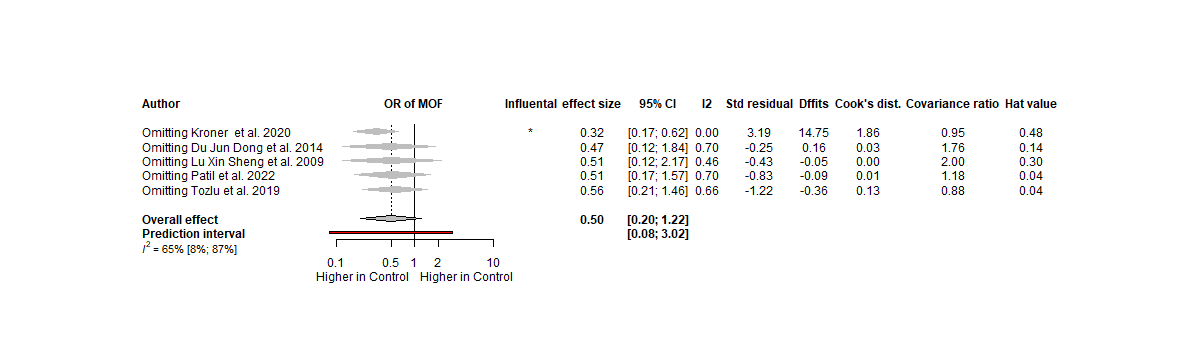


**Supplementary Figure 17**. Sensitivity analysis for multiple organ failure - Leave-one-out analysis: Kroner et. al is potential outlier

**
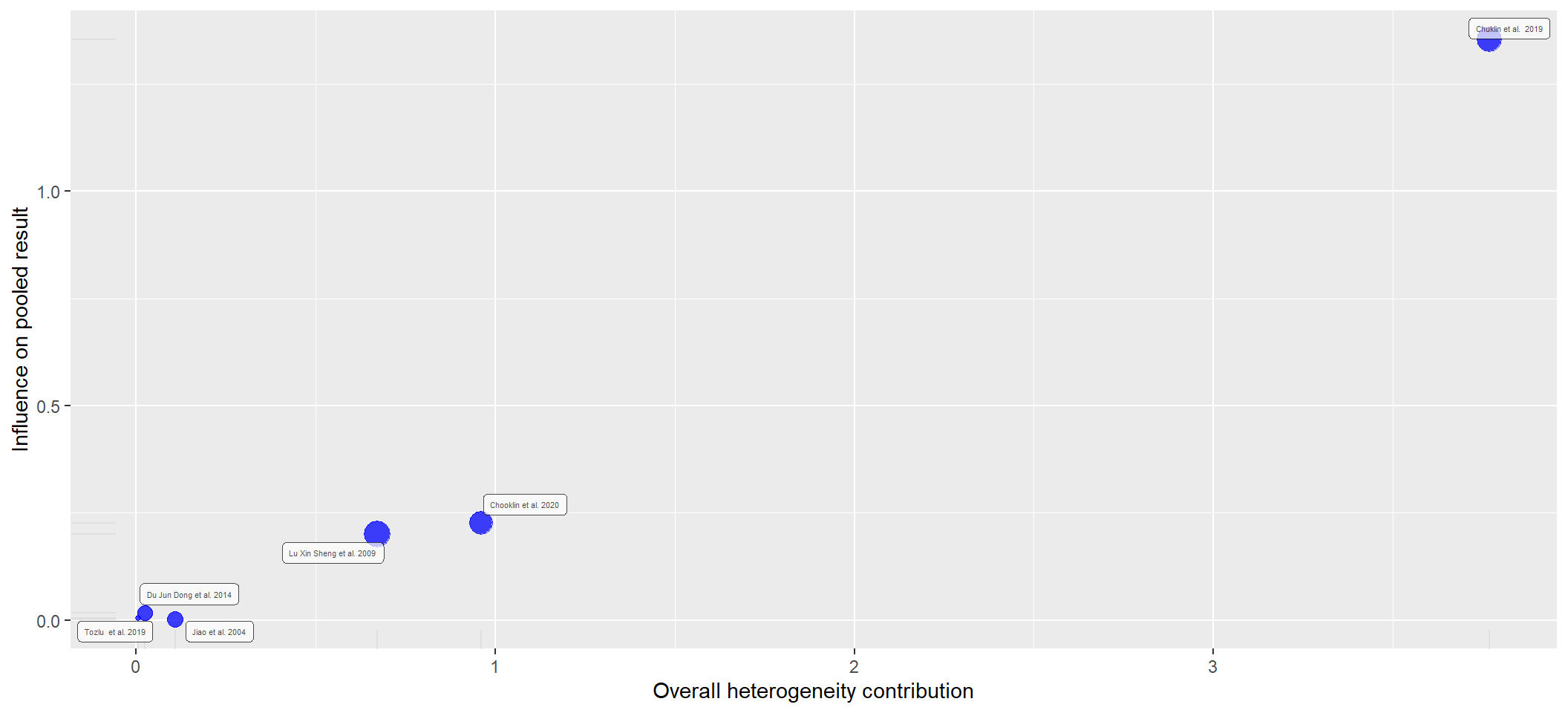
**

**Supplementary Figure 18**. Sensitivity analysis for need for endoscopic or surgical interventions - Baujat plot: Chuklin et. al is a potential outlier.


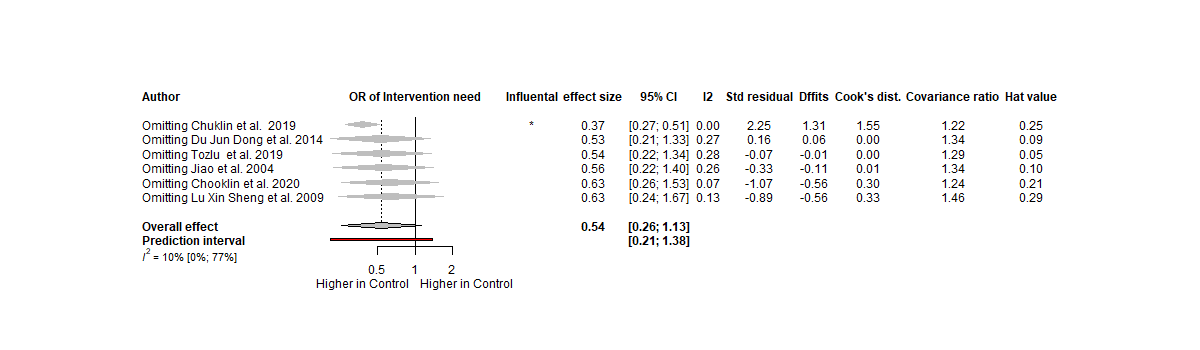


**Supplementary Figure 19**. Sensitivity analysis for need for endoscopic or surgical interventions- Leave-one-out analysis: Chuklin et. al is potential outlier

**
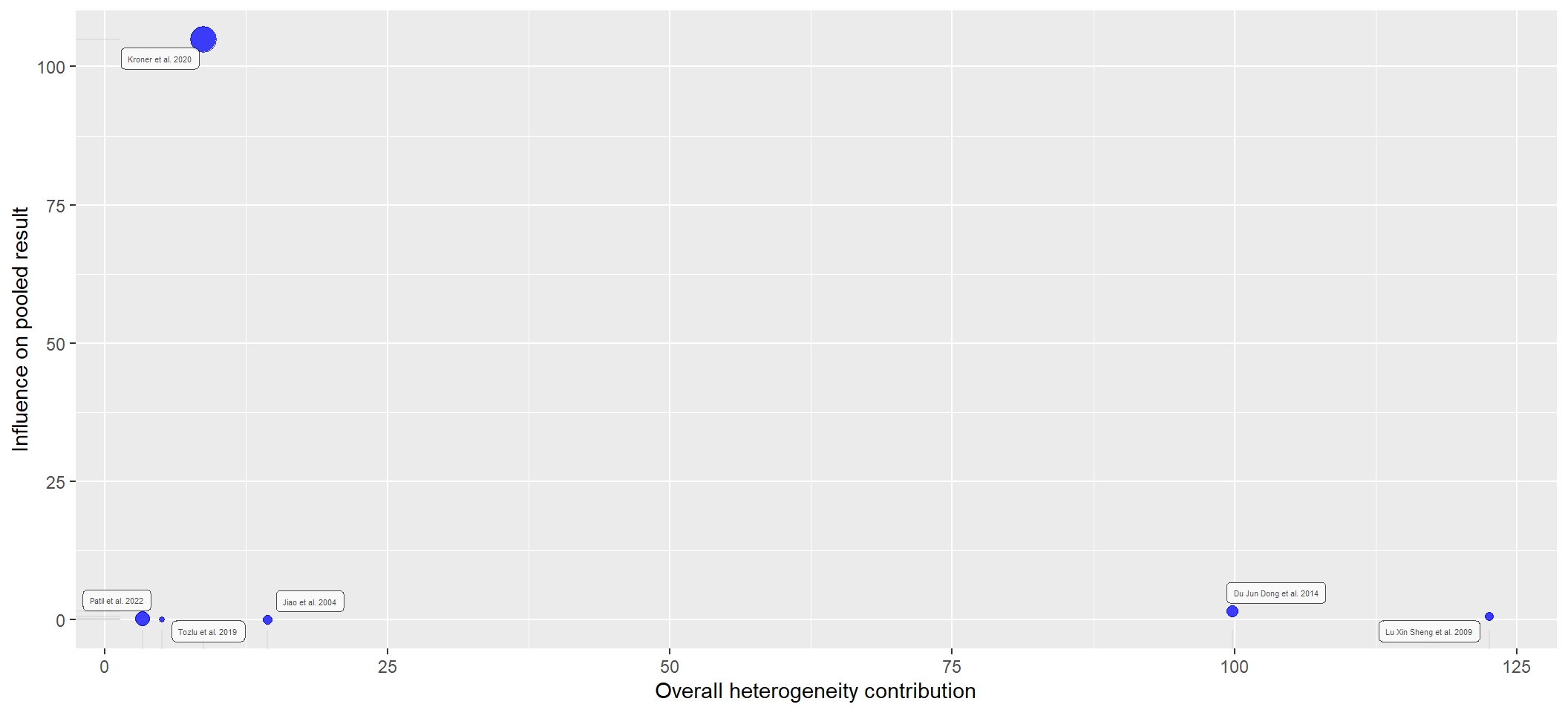
**

**Supplementary Figure 20**. Sensitivity analysis for need for length of hospital stay - Baujat plot

**
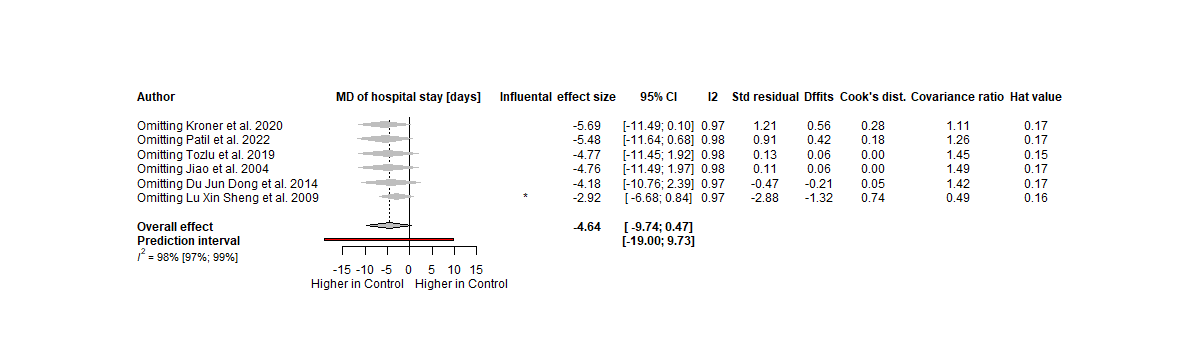
**

**Supplementary Figure 21**. Sensitivity analysis for length of hospital stay - Leave-one-out analysis: Kroner et al and Lu et al are potential outliers

**REFERENCE**

1. Mantel N, and William Haenszel. Statistical Aspects of the Analysis of Data From Retrospective Studies of Disease. JNCI: Journal of the National Cancer Institute. 1959;22(4):719–48.

2. Robins J, Sander Greenland, and Norman E. Breslow. A General Estimator for the Variance of the Mantel-Haenszel Odds Ratio. American Journal of Epidemiology. 1986:719-23.

3. Thompson SG, Rebecca M Turner, and David E Warn. Multilevel Models for Meta-Analysis, and Their Application to Absolute Risk Differences. Statistical Methods in Medical Research. 2001;10(6):375–92.

4. Cooper HM, Larry V. Hedges, and Jeff C. Valentine. The Handbook of Research Synthesis and Meta-Analysis. 2nd ed. New York: Russell Sage Foundation. 2009.

5. J. Sweeting M, Alexander J. Sutton, and Paul C. Lambert. What to Add to Nothing? Use and Avoidance of Continuity Corrections in Meta-Analysis of Sparse Data. Statistics in Medicine. 2004;23(9):1351–75.

6. Harrer M, Pim Cuijpers, Furukawa Toshi A, and David D Ebert. Doing Meta-Analysis With R: A Hands-On Guide. 1st ed. Boca Raton, FL; London: Chapman & Hall/CRC Press. 2021.

7. Viechtbauer W, and Mike W.-L. Cheung. Outlier and Influence Diagnostics for Meta-Analysis. Research Synthesis Methods. 2010;1 (2):112–25.

8. Harbord RM, Matthias Egger, and Jonathan A. C. Sterne. A Modified Test for Small-Study Effects in Meta-Analyses of Controlled Trials with Binary Endpoints. Statistics in Medicine. 2006;25 (20):3443–57.

9. Sterne JAC, A. J. Sutton, J. P. A. Ioannidis, N. Terrin, D. R. Jones, J. Lau, J. Carpenter, et al. Recommendations for Examining and Interpreting Funnel Plot Asymmetry in Meta-Analyses of Randomised Controlled Trials. BMJ. 2011;343:d4002–2.
